# Exploratory Study of Biomechanical Properties and Pain Sensitivity at Back-Shu Points

**DOI:** 10.3390/brainsci14080823

**Published:** 2024-08-16

**Authors:** Heeyoung Moon, Seoyoung Lee, Da-Eun Yoon, In-Seon Lee, Younbyoung Chae

**Affiliations:** 1 Department of Science in Korean Medicine, Graduate School, Kyung Hee University, Seoul 02447, Republic of Korea; mistymoon91@naver.com (H.M.); yoonda05@khu.ac.kr (D.-E.Y.); inseon.lee@khu.ac.kr (I.-S.L.); 2 Department of Behavioral Medicine, Faculty of Medicine, Institute of Basic Medical Sciences, University of Oslo, 0372 Oslo, Norway; seoyoung.lee@khu.ac.kr

**Keywords:** back-shu points, diagnosis, muscle stiffness, muscle tone, pain sensitivity

## Abstract

Objectives: Hypersensitive acupoints in specific body areas are associated with corresponding internal or visceral disorders. Back-shu points are clinically significant for the diagnosis of visceral organ disease, according to the biomechanical characteristics of the acupoints. In this study, we assessed the biomechanical characteristics and pain sensitivities of five back-shu points linked to five visceral organs in healthy participants. Methods: The study included 48 volunteer participants. A myotonometry was used to assess muscle tone and muscle stiffness at five back-shu points associated with visceral organs. Pressure was monitored using a microcontroller and a force sensor. Pain sensitivity was assessed in response to deep pressure pain produced by a constant force. Results: Substantial differences in muscle tone and stiffness were observed at the five back-shu points; muscle tone was highest at BL15, whereas muscle tone and muscle stiffness were lowest at BL23. Moreover, pain sensitivity was significantly different among the acupoints; pain sensitivity was highest at BL23. There was a significant negative correlation between muscle tone and pain sensitivity. Conclusions: We found significant differences in muscle tone, muscle stiffness, and pain sensitivity among five back-shu points associated with visceral organs, which may be attributable to anatomical variations at each point. Our findings suggest that differences at back-shu points should be considered to ensure the accurate diagnosis of visceral disease.

## 1. Introduction

Acupoints are certain points on the body that an acupuncture needle is inserted into to stimulate [1,2]. The majority of traditional acupoints are found on hyperirritable myofascial trigger points within taut bands of skeletal muscles or surrounding peripheral nerves directly influenced by non-pharmacological neuromodulation [3,4]. Acupoint sensitization is the term used to describe sensory hypersensitivity and functional plasticity in various disorders [5]. Cutaneous hypersensitivity in specific body areas may be a sign of pathological conditions in the corresponding visceral organ [6,7]. It has been demonstrated that certain acupoints on the skin exhibit various signs of cutaneous neurogenic inflammation, including increased pain sensitivity, through a viscero-somatic connection [8]. Acupoints can be thought of as dynamic, functional entities whose physiological characteristics (e.g., pain sensitivity) are associated with corresponding internal or visceral diseases [9]. When sensitized acupoints are stimulated, relevant conditions can be treated more successfully [10,11]. Thus, it is critical to identify hypersensitive acupoints in order to determine which acupoints are best for acupuncture treatment. However, numerous studies have examined whether pathologic visceral conditions alter the sensitivity of a particular acupoint to pain, despite a number of methodological flaws, such as inadequate reporting of the variables and experimental designs [12].

Back-shu points are closely associated with the function and health of internal organs. When utilized in diagnosis and treatment, these acupoints are thought to correspond to particular internal organs by regulating sympathetic and parasympathetic nervous system activity [13]. Back-shu points are used to treat visceral diseases because they are thought to carry *qi* to the internal organs [14]. These points are located 1.5 B-cun lateral to the inferior border of the corresponding spinous processes on the outer border of the erector spinae muscles [15]. Back-shu point needling has multiple effects on visceral organs [13]. A relationship exists between back-shu points and the distribution of segmental neurons in the spinal cord [13]. Somatosensory inputs from the skin and/or muscle are involved in the control of several autonomic activities through the viscerocutaneous reflex pathway; thus, the stimulation of back-shu points activates the associated segmental autonomic nerves [16,17]. Acupuncture at BL18, BL20, and BL22 is an effective treatment for gastrointestinal diseases due to spinal nerve compression, sympathetic nerve hyperactivity, and diaphragm connections [18]. Functional dyspepsia was treated with BL20 and BL21 acupoints (upper back region, back-shu point for spleen, and stomach, respectively), whereas irritable bowel syndrome was treated with BL23 and BL25 acupoints (lower back region, back-shu points for kidney and large intestine) [19]. Back-shu point treatment regulates the functions of visceral organs. In addition, these back-shu points are utilized to control the functions of internal organs along with front-mu points [20]. In both scientific and clinical contexts, combinations of front-mu and back-shu points have been widely used [14].

In addition to their importance in clinical treatments, back-shu points have been used to diagnose visceral organ diseases [13]. The palpation of sensitive back-shu points may produce positive reactions, such as relaxation, or negative reactions, such as discomfort, which can be used to identify the states of visceral organs [15]. Similar to skin afferents, visceral afferent nociceptors converge on the same pain projection neurons. The information from these two types of input is substantially combined in the spinal cord [13]. For the back-shu points, there is greater segmental organ correspondence at the deeper muscle layers. Visceral organ dysfunction can cause pain, hyperalgesia, tension, or irritation to a specific area of skin through the viscerocutaneous reflex [13]. For example, patients with chronic asthma showed specific changes in the body surface temperature at relevant back-shu points [21]. Considering the chemical characteristics and neural pathways, the BL23 acupoint (back-shu point for the kidney) and kidney have a close sensory and sympathetic relationship, suggesting that back-shu points control target organ activity through peripheral sensory and sympathetic pathways [22]. However, these acupoints’ diagnostic efficacies need to be rigorously assessed in carefully designed experiments.

Although back-shu points are widely used to diagnose visceral organ disorders, no studies have investigated whether the biomechanical properties and pain sensitivity vary among acupoint sites. The use of muscle tone, muscle stiffness, and pain sensitivity at various back-shu points to diagnose visceral organ disease assumes that there are no differences among acupoints in the same individual. However, differences in muscle tone, stiffness, and pain sensitivity at back-shu points may exist in healthy participants due to anatomical differences. Such differences, if not considered, may result in incorrect diagnoses. Here, we hypothesized that there were no differences in muscle tone, muscle stiffness, or pain sensitivity at back-shu points in the same individual. In the current study, we investigated the biomechanical properties and pain sensitivity at five visceral-associated back-shu points in healthy volunteers.

## 2. Materials and Methods

### 2.1. Participants

This study included 48 healthy volunteers who were recruited from Kyung Hee University, Seoul, Republic of Korea, through online advertisements. Participants were excluded if they had psychological or psychiatric disorders, vascular disorders, epilepsy, dementia, substance dependence, a previous history of brain surgery, cognitive impairments, skin pathologies, or sensory abnormalities; they were also excluded if they were traditional Korean medicine doctors or students majoring in traditional Korean medicine. Furthermore, participants with a body mass index <18.5 or >25 kg/m^2^ were excluded, in order to avoid including participants who were underweight or overweight. Participants were required to refrain from consuming alcohol, caffeine, or medication for 12 h before the experiment. All participants received a detailed explanation of the study and provided written informed consent prior to the commencement of the study. All experiments were conducted in accordance with guidelines issued by the human subjects committee and were approved by the institutional review board of Kyung Hee University, Seoul, Republic of Korea (approval number: KHSIRB-21-243).

### 2.2. Selection of Back-Shu Points

**Figure 1:**
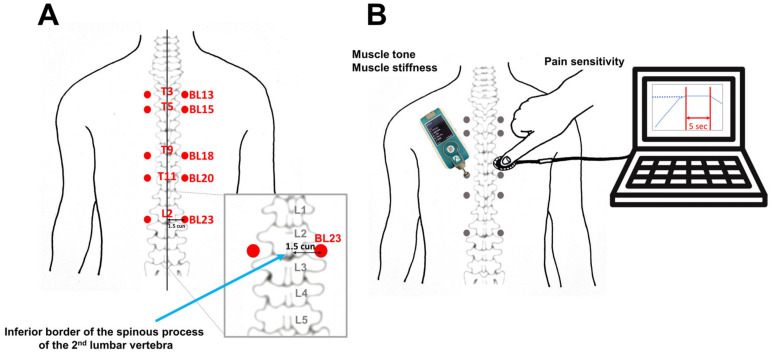
Experimental procedure. (**A**) Identification of five back-shu points on the back of the trunk associated with visceral organs. A traditional Korean medicine doctor identified back-shu points on the lateral border of the erector spinae muscles of each participant and marked the acupoints bilaterally with a dot. In order to identify back-shu points, practitioners first palpated the inferior border of the vertebra’s spinous process (marked with a blue arrow) and then located 1.5 B-cun lateral to the posterior median line. (**B**) Muscle properties and pain sensitivity were assessed at each back-shu point. Muscle tone and stiffness were measured using a Myoton PRO device (Myoton AS, Tallinn, Estonia), and pain sensitivity was assessed after the application of deep pressure with a constant force for 5 s. The degree of pressure was monitored using a force sensor and an Arduino UNO microcontroller (Arduino, New York, NY, USA). The force sensor was placed on the participant’s skin, and the assessor applied pressure to the sensor using the thumb. Real-time pressure was monitored using an Arduino UNO device and the Simulink software (version 2020a; MathWorks, Natick, MA, USA). After palpation pressure had been applied at each back-shu point, participants rated pain intensity on an 11-point numeric pain scale.

The participants were given an explanation of the study protocol. Five back-shu points associated with visceral organs were identified according to the World Health Organization standard acupoint locations (Figure 1A). BL13 (back-shu point for the lung) is located in the upper back region at the level of the inferior border of the spinous process of the third thoracic vertebra, 1.5 B-cun lateral to the posterior median line. BL15 (back-shu point for the heart) is located in the upper back region at the level of the inferior border of the spinous process of the fifth thoracic vertebra, 1.5 B-cun lateral to the posterior median line. BL18 (back-shu point for the liver) is located in the upper back region at the level of the inferior border of the spinous process of the ninth thoracic vertebra, 1.5 B-cun lateral to the posterior median line. BL20 (back-shu point for the spleen) is located in the upper back region at the level of the inferior border of the spinous process of the 11th thoracic vertebra, 1.5 B-cun lateral to the posterior median line. BL23 (back-shu point for the kidney) is located in the lumbar region at the level of the inferior border of the spinous process of the second lumbar vertebra, 1.5 B-cun lateral to the posterior median line.

In order to identify back-shu points, practitioners first palpated the inferior border of the vertebra’s spinous process and then located 1.5 B-cun lateral to the posterior median line. The back-shu points were marked bilaterally with a medical pen by a well-trained traditional Korean medicine doctor who was licensed to practice acupuncture (H.M.). Back-shu points are typically identified by their palpable structure or by a lower pain threshold in response to pressure pain. In order to quantify the biomechanical characteristics of the muscles, we analyzed the muscle tone and stiffness of back-shu points. We also assessed the pain sensitivity of back-shu points while applying deep pressure.

### 2.3. Measurement of Biomechanical Properties

Participants were asked to lie on a bed in the prone position. The traditional Korean medicine doctor measured biomechanical properties, including muscle tone and muscle stiffness, at the acupoints using a Myoton PRO device (Figure 1B). The device delivered a weak force (0.4 N) for 15 ms, which damped natural oscillation in the underlying tissue. The Myoton PRO provides an objective, non-invasive measure of the tissue response and viscoelastic properties [23]. Muscle tone (Hz) is the natural oscillation frequency, defined as the intrinsic state of resting tension without active contraction [24]. Muscle stiffness (N/m) is a measure of the muscle’s resistance to an applied deforming force. Myotonometry is a reliable and valid instrument for assessing the biomechanical properties of muscles and tendons [25,26].

Muscle tone and stiffness were measured at the five back-shu points in all participants. As the assessor performed the measurement, the researcher who identified the back-shu point noted the muscle tone and stiffness displayed on the Myoton PRO screen. We standardized the process so that each assessment was conducted in the same sequence. An Excel (Microsoft Excel 2016) random number generator was used to randomly allocate each participant to either the descending or ascending order groups. The biomechanical properties were assessed from BL13 to BL23 in half of the participants and from BL23 to BL13 in the other half.

The muscle tone and stiffness values for each back-shu point were averaged across the left and right sides. We calculated the correlation coefficient values of muscle tone between the left and right side back-shu points before averaging the two sides of the back-shu points. High correlation coefficient values were observed at five distinct areas on the left and right side of the back-shu points (BL13: r = 0.774, *p* < 0.001, BL15: r = 0.747, *p* < 0.001, BL18: r = 0.815, *p* < 0.001, BL20: r = 0.812, *p* < 0.001, BL23: r = 0.840, *p* < 0.001). We also calculated the correlation coefficient values of muscle stiffness between the left and right back-shu points before averaging the two sides of the back-shu points. High correlation coefficient values were observed at five distinct areas on the left and right sides of the back-shu points (BL13: r = 0.756, *p* < 0.001, BL15: r = 0.703, *p* < 0.001, BL18: r = 0.769, *p* < 0.001, BL20: r = 0.822, *p* < 0.001, BL23: r = 0.777, *p* < 0.001). These data can ensure the test–retest reliability of measurements of muscle tone and stiffness.

### 2.4. Measurement of Pain Sensitivity

Pain sensitivity was assessed in response to a 5-second duration of deep pressure pain produced by the assessor’s right thumb with a constant force. Pressure was monitored using a force sensor (FSR402, Interlink Electronics, Camarillo, CA, USA) and an Arduino UNO microcontroller (Arduino, New York, NY, USA). The thin sensor of the force-sensitive resistor was placed between the participant’s skin, marked with a cross, and the assessor’s thumb to monitor real-time pressure using the Arduino UNO device and Simulink software (version 2020a; MathWorks, Natick, MA, USA, Figure 1B) [27]. Our previous study addressed in detail the validation and calibration of force sensors for pressure monitoring [27].

After pressure had been applied at each back-shu point, participants rated pain using an 11-point numeric pain rating scale that ranged from “0”, representing the least severe pain (“No pain”), to “10”, representing the most severe pain (“Pain as bad as you can imagine”). To determine the sensitivities of the sites, each participant was asked to express their pain verbally right after compression.

The pain level at each back-shu point was averaged across the left and right sides. We calculated the correlation coefficient values of pain ratings between the left and right back-shu points before averaging the two sides of the back-shu points. High correlation coefficient values were observed at five distinct areas on the left and right sides of the back-shu points (BL13: r = 0.818, *p* < 0.001, BL15: r = 0.707, *p* < 0.001, BL18: r = 0.880, *p* < 0.001, BL20: r = 0.603, *p* < 0.001, BL23: r = 0.720, *p* < 0.001). These data can ensure the test–retest reliability of the measurement of pain ratings.

### 2.5. Statistical Analysis

Significant differences in muscle tone, muscle stiffness, and pain sensitivity among back-shu points were assessed via one-way analysis of variance (ANOVA), followed by the Tukey post hoc test. Pearson’s correlation analysis was used to determine the relationship between muscle tone and pain sensitivity. Statistical analyses were performed using the Jamovi software (version 0.9; http://www.jamovi.org (accessed on 10 July 2023)). All values are shown as means ± standard errors. *p* values < 0.05 were considered statistically significant.

## 3. Results

### 3.1. Baseline Characteristics of Participants

A total of 48 participants (age 22.5 ± 2.3) participated in this study. They included 26 females and 22 males in this study. The body mass index was 21.4 ± 1.6.

### 3.2. Biomechanical Properties among Back-Shu Points

**Figure 2:**
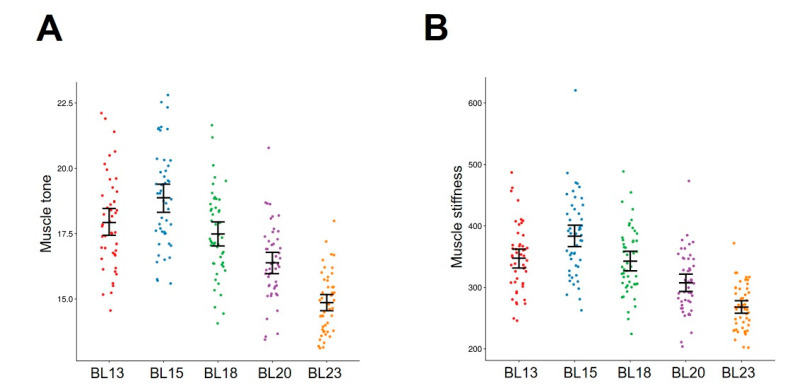
Biomechanical properties of the back-shu points. (**A**) Significant differences in muscle tone were observed among the back-shu points (F = 54.8, *p* < 0.001). Muscle tone was significantly lower at the BL23 acupoint than at the other back-shu points. (**B**) Muscle stiffness was significantly different among back-shu points (F = 39.36, *p* < 0.001). Muscle stiffness was significantly lower at the BL23 acupoint than at the other back-shu points.

Muscle tone was significantly different among back-shu points (F = 54.8, *p* < 0.001). Post hoc analysis revealed that muscle tone was significantly lower at the BL23 acupoint than at the other back-shu points (BL23: 14.9 ± 0.2 vs. BL13: 17.9 ± 0.3, BL15: 18.9 ± 0.3, BL18: 17.5 ± 0.2, BL20: 16.4 ± 0.2, paired *t* test, *p* < 0.01, Bonferroni corrected) (Figure 2A).

Muscle stiffness was also significantly different among back-shu points (F = 39.36, *p* < 0.001). Post hoc analysis revealed that muscle stiffness was significantly lower at the BL23 acupoint than at the other back-shu points (BL23: 267.9 ± 5.3 vs. BL13: 347.5 ± 7.9, BL15: 383.2 ± 9.6, BL18: 342.7 ± 7.9, BL20: 307.4 ± 7.3, paired *t* test, *p* < 0.01, Bonferroni corrected) (Figure 2B).

### 3.3. Pain Sensitivity among Back-Shu Points

**Figure 3:**
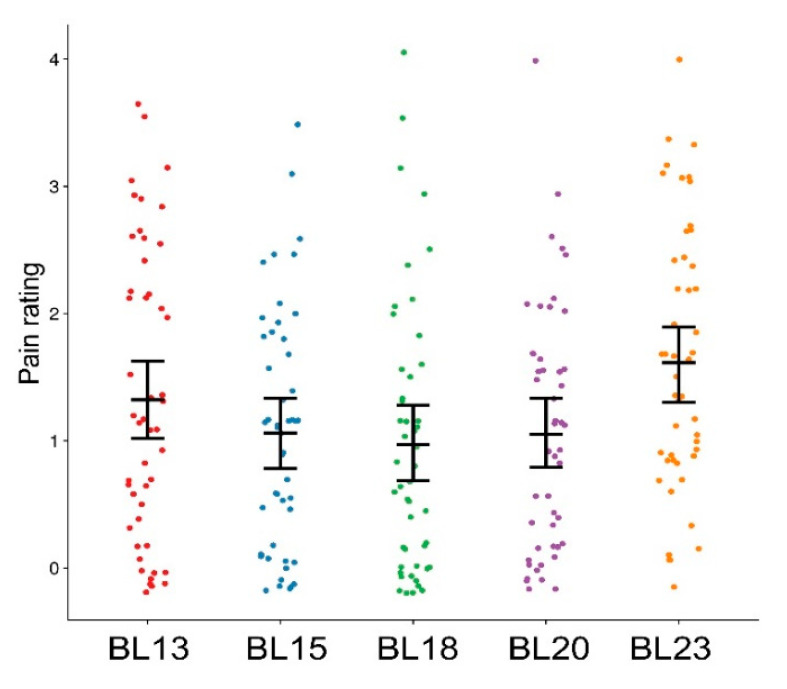
Pain sensitivity at back-shu points. Significant differences in pain sensitivity were observed among back-shu points (F = 39.36, *p* < 0.001). Pain sensitivity was significantly higher at the BL23 acupoint than at the BL18 acupoint.

Pain sensitivity was significantly different among back-shu points (F = 39.36, *p* < 0.001). Post hoc analysis revealed that pain sensitivity was significantly greater at the BL23 acupoint than at the BL18 acupoint (BL23: 1.6 ± 0.2 vs. BL18: 1.0 ± 0.2) (Figure 3).

### 3.4. Correlation between Muscle Properties and Pain Sensitivity

We found a negative correlation between muscle tone and pain sensitivity (r = −0.172, *p* < 0.01).

## 4. Discussion

We found significant differences in the biomechanical characteristics of muscle tone and stiffness, as well as pain sensitivity, among five back-shu points associated with visceral organs. Moreover, we found a negative correlation between muscle tone and pain sensitivity. Anatomical differences across regions of the back may explain the differences in biomechanical properties and pain sensitivity among acupoints. Clinicians should consider these differences in back-shu points to ensure accurate diagnoses.

The five back-shu points assessed in this study are located 1.5 B-cun lateral to the inferior border of the corresponding spinous processes on the outer border of the erector spinae muscles. Only healthy volunteers were enrolled in our study, and we did not examine biomechanical characteristics or pain sensitivity in patients with visceral disease. The assessment of visceral organ function based on muscle tone, muscle stiffness, and pain sensitivity at back-shu points assumes that there are no differences among acupoints in the same individual. However, we observed differences in biomechanical properties and pain sensitivity among the back-shu points. Specifically, muscle tone and muscle stiffness were lower, while pain sensitivity was higher, at the BL23 acupoint (kidney; lower trunk) than in the other back-shu points (BL13, BL15, BL18, and BL20; upper trunk). Healthcare professionals unaware of these differences may incorrectly diagnose their patients with kidney dysfunction. The biomechanical and pain sensitivity differences at these acupoints must be considered to ensure accurate diagnoses.

Numerous studies have attempted to examine whether pain sensitivity at a particular acupoint can be changed in pathological visceral conditions [12]. Nevertheless, a small study was performed to investigate these changes at back-shu points. A previous study demonstrated that patients with chronic asthma exhibited particular changes in the body surface temperature at relevant back-shu points [21]. On the other hand, there was no study that investigated the biomechanical characteristics and pain sensitivity of back-shu points. We presented data on the biomechanical characteristics and pain sensitivity of back-shu sites in healthy subjects in the current investigation. To achieve a correct diagnosis, practitioners must take into account the variations in biomechanical and pain sensitivity at back-shu points when examining their patients with biomechanical properties and pain sensitivity. In the future, it will be important to compare these measurements with those of patients who have disorders of the visceral organs.

Myofascial trigger points are characterized by hyperirritable points located in the taut band and exhibit a lower pressure pain threshold and higher muscle stiffness compared with adjacent muscle areas [27,28]. In the current study, on the other hand, we found a negative correlation between muscle tone and pain sensitivity at five different back-shu points, which suggests that back-shu points with lower muscle tone had a tendency toward higher pain. Our findings suggest that back-shu points showed different characteristics from myofascial trigger points. Thus, it is considered that the biomechanical features and pain sensitivity of back-shu points might be more closely associated with the functions of visceral organs than the problems of musculoskeletal diseases. However, further research will be required.

Various anatomical characteristics may explain the differences found at back-shu points. The erector spinae muscles are covered in multiple layers of muscle; the superficial layer of the back muscles includes the latissimus dorsi, rhomboid, levator scapulae, and trapezius muscles. The latissimus dorsi muscle is a broad, flat muscle that occupies the majority of the lower posterior thorax, whereas the trapezius muscle is a large, triangular, paired muscle located on the posterior aspect of the neck and the upper posterior thorax. The trapezius muscle is specifically attached to the spinous processes of the C7–T12 vertebrae, the nuchal ligament, and the medial third of the superior nuchal line. The latissimus dorsi is attached to the supraspinous ligament, the lumbar and sacral spinous processes (T6 to S5 levels), and the thoracolumbar fascia [29]. A large amount of the fascia surrounding the latissimus dorsi muscle is presumably responsible for the reduced muscular tone and muscular stiffness, as well as increased pain sensitivity, at the BL23 acupoint in the lower back. Further research is needed to fully understand the variations in biomechanical characteristics and pain sensitivity among back-shu points. Furthermore, ultrasonography will be required to visualize the underlying deep structure of the back-shu points.

It is noteworthy that the myotonometry was used to measure the degree of tenderness of back-shu points rather than manual palpation. According to a recent systematic analysis of human acupoint sensitivity, manual palpations employing the investigators’ fingertip pressure were utilized to evaluate the amount of tenderness surrounding acupoints [12]. Three studies that examined the occurrence of tenderness at acupoints discovered that clinical populations had higher rates of tenderness incidence [12]. Even though manual palpation is frequently employed in clinical settings, the investigators may have used varying degrees of force, which could have led to biased results. To evaluate the biomechanical characteristics of the back-shu points objectively, we employed myotonometers to determine the stiffness and tone of the muscles in the current investigation. This device is a relatively novel tool to measure the biomechanical and viscoelastic properties of palpable musculotendinous structures [30]. In our previous study, we found that myofascial trigger points, which are hyperirritable areas, had more muscle stiffness when utilizing this device [27]. Thus, it is expected that myotonometry could provide an objective tool for evaluating the degree of tenderness surrounding acupoints.

Our study had several limitations. First, it only included young, healthy volunteers. Furthermore, assessments of muscle tone, muscle stiffness, and pain sensitivity were restricted to five back-shu points associated with visceral functions. Further studies comparing biomechanical properties and pain sensitivity at back-shu points in healthy volunteers and patients with specific visceral diseases are needed to determine the utility of these measures for diagnosing visceral organ dysfunction. Second, pain sensitivity was assessed as the response to pressure pain from constant manual force produced by the assessor. We used a force sensor to record pressure; however, clinicians in real-world clinics may apply different forces at various acupoints. It is important to use a well-controlled robotic device to apply fixed pressure at the back-shu points. Third, we did not perform physical examinations, medical imaging diagnostics, or rule out the presence of osteochondrosis with radicular syndrome, intervertebral hernias, or protrusions, which could have an impact on the tone, stiffness, and sensitivity to pain of the back muscles. Furthermore, we did not evaluate the skin temperature or other kinds of pain sensitivity around the back-shu point. A variety of pain measures, such as mechanical and thermal pain, should be used to assess pain sensitivity at the back-shu points.

In summary, our study revealed substantial differences in muscle tone, muscle stiffness, and pain sensitivity at five back-shu points associated with visceral organs. Due to underlying structural variations, biomechanical properties and pain sensitivity may vary among the locations. It is important to consider these differences at back-shu points to ensure accurate diagnoses.

## Data Availability

The original contributions presented in the study are included in the article, further inquiries can be directed to the corresponding author.
